# Prevalence of multidrug-resistant coagulase-positive staphylococci in canine and feline dermatological patients over a 10-year period: a retrospective study

**DOI:** 10.1099/mic.0.001300

**Published:** 2023-02-14

**Authors:** Mikaela Burke, Domenico Santoro

**Affiliations:** ^1^​ Department of Small Animal Clinical Sciences, College of Veterinary Medicine, University of Florida, 2015 SW 16th Ave Gainesville, FL 32610, USA

**Keywords:** coagulase-positive staphylococci, dermatology, multidrug resistance, dogs, cats

## Abstract

Coagulase-positive staphylococci (CPS) are common cutaneous pathogens often requiring multiple courses of antibiotics, which may facilitate selection for methicillin-resistant (MR) and/or multidrug-resistant (MDR) strains. To determine the prevalence of canine and feline MR/MDR CPS associated with skin diseases, medical records were retrospectively searched from April 2010 to April 2020. Pets with at least one positive culture for CPS were selected. Age, sex, antimicrobial sensitivity, previous history of antimicrobial/immunomodulatory medications and methicillin resistance/multidrug resistance status were recorded. *Staphylococcus pseudintermedius* (SP) (575/748) and *Staphylococcus schleiferi* (SS) (159/748) in dogs, and *Staphylococcus aureus* (12/22) in cats, were the most common CPS isolated. Three hundred and twenty-three out of 575 isolates were MR-SP (56.2 %), 304/575 were MDR-SP (52.8 %), 100/159 were MR-SS (62.9 %) and 71/159 were MDR-SS (44.6 %). A trend analysis showed a significant increase of resistance to oxacillin and chloramphenicol for *S. pseudintermedius* (r=0.86, 0.8; *P*=0.0007, 0.0034, respectively). Major risk factors for MDR-SP included oxacillin resistance (OR: 3; 95 % CI: 1.4–6.5; *P=*0.0044), positivity for PBP2a (OR: 2.3; 95 % CI: 1–5; *P=*0.031) and use of antibiotics in the previous year (OR: 2.8; 95 % CI: 1.3–5.8; *P*=0.0071). Oxacillin resistance was identified as a major risk factor for MDR-SS (OR: 8.8; 95 % CI: 3.6–21.1; *P<*0.0001). These results confirmed the widespread presence of MR/MDR CPS in referred dermatological patients. Judicious antibiotic use, surveillance for MR/MDR infections and consideration of alternative therapies are crucial in mitigating the development of resistant strains.

## Introduction

Antimicrobial resistance is constantly expanding across many areas of medicine. In dermatology, chronic cutaneous infections caused by methicillin-resistant (MR) and/or multidrug-resistant (MDR) strains of staphylococci present a significant problem in small animal practice. Pan-susceptible staphylococci are becoming less common [1, 2]. Additionally, the degree of resistance among coagulase-positive staphylococci (CPS) can be increased by the isolation of coagulase-negative staphylococci, which are generally considered to exhibit low pathogenicity. They may harbour resistance determinants and potentially serve as reservoirs that may convey these determinants to more pathogenic organisms [3].

Methicillin-resistant staphylococci (coagulase-positive or -negative) are naturally resistant to β-lactam antibiotics, including penicillins, cephalosporins and carbapenems. This is due to the presence of the *SCCmec* gene cluster, which transcribes an altered penicillin-binding protein (PBP2a) in these strains [4, 5]. Methicillin-resistant staphylococci are likely to acquire resistance to other antibacterial agents and become MDR [6]. As the treatment options for MDR infections become limited, practitioners often resort to antibiotics such as rifampin and chloramphenicol, which can have adverse side effects for the patient (e.g. liver toxicity, bone marrow suppression, peripheral neuropathy) and/or the owners (e.g. aplastic anaemia in people with chloramphenicol) [7]. The potential transfer of genetic elements between bacterial species has raised concerns regarding the zoonotic potential of these pathogens [8, 9].

*Staphylococcus pseudintermedius* (SP), *Staphylococcus schleiferi* (SS) and *Staphylococcus aureus* (SA) are some of the most commonly cultured species of staphylococci from cutaneous bacterial infections in companion animals [10]. *S. schleiferi* has not been widely researched in the veterinary literature, but frequently contributes to skin and ear infections in dogs [11]. *S. aureus *, a prominent cause of staphylococcal infections in humans, is uncommonly reported in dogs and cats. *S. pseudintermedius* is instead the most common cause of staphylococcal infections in these species [5, 6]. The coagulase-positive *S. pseudintermedius* is a commensal, opportunistic pathogen harboured by the skin and mucous membranes of dogs and cats. It has been suggested that disturbances in the body’s microbiota secondary to a disease status, such as atopic dermatitis, or recent exposure to antibiotics can select resistant bacterial infections [4, 6]. Indeed, recurrent antibiotic use may increase the selection pressure for MDR bacterial strains. Furthermore, the high incidence of methicillin resistance in staphylococci highlights concerns (zoonotic) over the transmission of resistant genes between different populations of the organisms [6].

A clear distribution of MR and/or MDR staphylococcal infections in dogs and cats has not been identified. Many studies have been published indicating a potential geographical distribution of antibiotic resistance in veterinary medicine [12–15]. Epidemiological studies have shown how the percentage of MR-SP and MDR-SP can vary dramatically in different parts of the world, ranging from 14 % in Finland to 66.5 % in Japan [12–14]. However, studies have shown a low prevalence, ranging from 2.5–5.1 %, of MDR in dogs in the UK [16]. Even less information is present on the prevalence of MR-SP and MDR-SP in cats with pyoderma. Only one study, published in 2014 in Germany, reported a prevalence of 10.7 % of cats with MR-SP (out of 283 MR-SP culture submissions) [17].

Likewise, not many studies have analysed the potential risk factors associated with methicillin resistance in either dogs or cats. A German study looked at the presence of risk factors associated with MR-SP in dogs and cats [17]. The study concluded that dogs and cats that had been hospitalized or visited veterinary clinics and that had received topical ear medication or glucocorticoids were at higher risk of MR-SP infection. However, no data have been reported on the risk factors for MDR in companion animals.

The prominence of resistance has been shown to vary depending upon many factors (e.g. geographical area or primary versus referral units) and potentially increased over time [2, 3, 16]. More studies are needed to better identify epidemiological factors that contribute to increased levels of multidrug resistance in cutaneous bacterial infections in dogs and cats. Consequently, the main goals of this study were (1) to determine whether the incidence of cutaneous MR and MDR CPS in dogs and cats seen by a tertiary dermatology clinic has increased over the past 10 years and (2) to identify risk factors associated with multidrug resistance status.

## Methods

### Study population

Medical records of canine and feline patients seen by the dermatology service at the University of Florida – College of Veterinary Medicine from April 2010 through April 2020 were retrospectively searched in the electronic medical system Cornerstone (IDEXX, Westbrook, ME, USA). Dermatological patients with at least one positive skin culture for CPS (* S. pseudintermedius* or *S. aureus *) or *S. schleiferi* (currently identified as *S. coagulans* and *S. schleiferi *) were selected. For each patient, the following data were collected: age, sex, breed, year of culture, number of bacterial cultures, species of bacteria, antimicrobial sensitivity, previous use and timeline of antibiotics and immunomodulatory drugs, as well as methicillin resistance/multidrug resistance status.

Isolates were identified by the clinical microbiology laboratory at the authors’ institution. Each isolate was considered non-susceptible to an antibacterial agent when listed as resistant (R) or intermediate (I) on the culture and sensitivity report. The no interpretation (NI) data were removed from the analysis. The NI interpretation was mainly based on lack of guidelines from the Clinical and Laboratory Standards Institute (CLSI) for the specific antibiotics, organism and site. The CPS isolates were processed for routine bacterial culture and sensitivity as per CLSI guidelines [18–21]. Each isolate was identified, based on biochemical reactions, using the Trek Sensititre (TREK Diagnostic Systems, Inc., Cleveland, OH, USA) automated system. The MIC was measured by the same system using CLSI guidelines active at the time of culture [18–21].

The methicillin resistance status for each isolate was confirmed via resistance to oxacillin (OXA) and confirmed via agglutination test for the penicillin binding protein 2 a (PBP2a) (Mast-Group, Liverpool, UK). For *S. aureus* isolates, cefoxitin was also evaluated for methicillin resistance status. Multidrug resistance was defined as acquired resistance to at least one agent in three or more antimicrobial classes [7].

Classes of antibiotics tested included β-lactams (amoxicillin/clavulanic acid, ampicillin, cefazolin, cefpodoxime, ceftiofur, cephalothin, imipenem, oxacillin), lincosamides (clindamycin), macrolides (erythromycin), tetracyclines (tetracycline, doxycycline, minocycline), aminoglycosides (gentamycin and amikacin), fluoroquinolones (enrofloxacin, marbofloxacin, pradofloxacin), phenicols (chloramphenicol), rifampin and sulfonamides [trimethoprim/sulfamethoxazole (TMS)].

### Statistical analysis

As a result of the low number of feline cases, epidemiological analysis was only performed on canine isolates. For feline patients, data were only reported descriptively.

Descriptive statistics for both dogs and cats were performed on selected variables (age, sex, methicillin resistance, multidrug resistance and number of classes of antibiotic in MDR isolates). The variable ‘age’ underwent to normality testing via Shapiro–Wilk test (α=0.05).

For canine isolates, conditional logistic regression was used to assess the association between multidrug resistance and the other variables analysed. Predictor variables were selected using a stepwise selection. Odd ratios (ORs) and their 95 % confidence intervals (CIs) were reported. The ORs were used as an epidemiological measure of association between a factor status [i.e. methicillin resistance (OXA and PBP2a), age at the time of culture, gender, number of classes of antibiotic resistance, use of antibiotic and anti-inflammatory medication] and risk of multidrug resistance. An OR of one indicated a lack of association between a particular factor and multidrug resistance. The greater the departure from one, the stronger the association between the factor and multidrug resistance. The variables for age (by using the median as a cutoff point) and number of classes of antibiotics (less than or greater than/equal to three) were dichotomized to facilitate the interpretation of the OR.

In addition, the Kruskal–Wallis test was used to compare the prevalence of MDR and MR staphylococci over time from 2010 to 2020 in dogs. Dunn’s multiple comparison test was used for post-hoc analysis. The Pearson or the Spearman correlation test was used to correlate the level of antibiotic resistance of each antimicrobial with time (trend analysis) [22, 23].

All hypotheses tested were considered significant when a *P* value <0.05 was achieved. All statistical analyses were performed using Prism 9.1.1 statistical software (GraphPad Software, Inc., La Jolla, CA, USA).

## Results

### Dogs

#### S. pseudintermedius

A total of 575 canine medical records with at least 1 positive culture for *S. pseudintermedius *, were retrieved. Of the dogs cultured, 332 were male and 243 were female. The age at the time of culture had an overall median of 6 years (range: 0.16–17 years). Data on the PBP2a were available for 495 isolates (86%), whereas all the isolates (575/575 – 100 %) were tested for OXA. A total of 301/575 (52.3 %) cultures had more than 1 organism isolated. A total of 512/575 (89 %) and 380/560 (67.8 %) records indicated exposure to a systemic antibiotic or an anti-inflammatory medication (i.e. glucocorticosteroids, oclacitinib, cyclosporine, lokivetmab), respectively, in the year prior to culture.

The percentage of methicillin resistance varied based on the detection of the presence of PBP2a or OXA resistance alone. In fact, 318/495 (55.3 %) isolates were positive by the PBP2a agglutination test and 323/575 (56.2 %) demonstrated OXA resistance (Table S1, available in the online version of this article). A total of 30/495 (6 %) of the isolates tested for both PBP2a and OXA had discordant results. Of the 30 isolates, 13 were positive for OXA but negative for PBP2a. Seventeen of the 30 isolates were negative for OXA but positive for PBP2a. A very strong correlation between PBP2a and OXA (r: 0.86; *P*<0.0001) was found for canine *S. pseudintermedius* isolates. When resistance to more than three classes of antibiotics was considered, 304/575 (52.8 %) isolates were MDR-SP, with an overall resistance to 6 (median) classes of antibiotics (range: 3–8) (Table S1). Over the time period studied the percentage of resistance to other classes of antibiotics ranged from 13±11 % (mean±standard deviation) for chloramphenicol to 52±11 % for lincosamides (Table 1). Rifampin was the exception, with only one isolate determined to be resistant over the 10-year period.

**Table 1.** Percentages of resistance to the different classes of antibiotic analysed for the 575 canine isolates of *Staphylococcus pseudintermedius
*

	MR-SP (PBP2a)	MR-SP (OXA)	MDR-SP	Macrolides	Lincosamides	Phenicols	Tetracyclines	Aminoglycosides	Fluoroquinolones	TMS	Rifampin
2020 (*n*=20)	72	80	65	30	63	26	58	18	44	55	0
2019 (*n*=81)	82	79	63	30	59	38	59	27	59	52	0
2018 *(n*=73)	63	61	47	24	48	17	65	17	79	48	0
2017 (*n*=66)	60	64	59	30	59	9	40	22	51	50	11
2016 (*n*=59)	68	66	42	22	44	12	0	12	36	42	0
2015 (*n*=81)	57	45	49	17	35	10	0	18	22	33	0
2014 (*n*=90)	48	53	48	22	43	17	0	28	33	43	0
2013 (*n*=13)	36	31	62	47	62	8	33	46	50	69	0
2012 (*n*=10)	86	60	70	25	50	0	0	25	30	40	0
2011 (*n*=25)	73	36	68	34	68	8	0	32	56	54	0
2010 (*n*=57)	87	30	42	18	37	2	30	22	22	35	0

MDR-SP, multidrug-resistant SP; MR-SP, methicillin-resistant SP; OXA, oxacillin; PBP2a, penicillin-binding protein 2 a; SP, *Staphylococcus pseudintermedius *; TMS, trimethoprim/sulfamethoxazole.

**Fig. 1.**
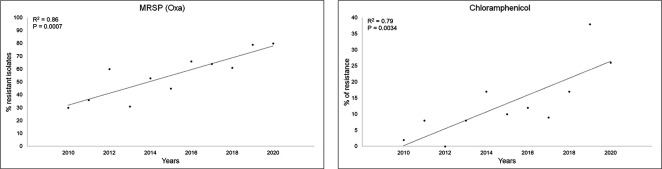
Percentage of resistance over time for oxacillin and chloramphenicol among the canine isolates of *Staphylococcus pseudintermedius *.

Compared with 2010, a significant increase in OXA resistance for *S. pseudintermedius* was seen in 2014 (*P*=0.025), 2015 (*P*=0.0045), 2016 (*P*=0.0003), 2017 (*P*=0.0007), 2018 (*P*=0.0022), 2019 (*P*<0.0001) and 2020 (*P*=0.0005). The percentage of MR-SP (OXA) increased from 28–80 % over the past 10 years. An increase in PBP2a or multidrug resistance status was not seen for *S. pseudintermedius *. When the relationship of resistance prevalence with time was assessed, a strong positive correlation was seen between time and methicillin resistance (OXA) status (r=0.86; *P*=0.0007). This was also seen with time and resistance to chloramphenicol (r=0.8; *P*=0.0034) (Figs 1 and S1).

In the multivariate analysis, the odds of having MDR-SP were three times higher in dogs with MR-SP (OXA) (OR: 3; 95 % CI: 1.4–6.5; *P=*0.0044), about two times higher in dogs with MR-SP (PBP2a) (OR: 2.3; 95 % CI: 1–5; *P=*0.031) and about three times higher in dogs with a history of antibiotic use in the previous year (OR: 2.8; 95 % CI: 1.3–5.8; *P*=0.0071) when compared to dogs with non-MR-SP or no history of previous use of antibiotics.

#### S. schleiferi

A total of 159 canine medical records with at least 1 positive culture for *S. schleiferi* were retrieved. Of the dogs cultured, 94 were male and 64 were female. The age at the time of culture had an overall median of 4.5 years (range: 1–9 years). Data on the PBP2a were available for 138 isolates (87 %), whereas OXA was tested for all the isolates (159/159–100 %) (Table S1). A total of 117/158 (74 %) cultures had more than 1 organism isolated. A total of 147/158 (93 %) and 119/145 (82 %) records indicated exposure to a systemic antibiotic or an anti-inflammatory medication (i.e. glucocorticosteroids, oclacitinib, cyclosporine, lokivetmab), respectively, in the year prior to culture.

Similar to *S. pseudintermedius *, for *S. schleiferi* the percentage of methicillin resistance also varied slightly based on the detection of the presence of PBP2a or OXA resistance alone. In fact, 79/139 (56.8 %) isolates were positive for the PBP2a agglutination test and 100/159 (62.9 %) showed OXA resistance. A total of 10/139 (0.7 %) of the isolates tested for both PBP2a and OXA had discordant results. Nine isolates were positive for OXA but PBP2a-negative and one isolate was negative for OXA but positive for PBP2a. A very strong correlation between PBP2a and OXA (r: 0.82; *P*<0.0001) was found for canine *S. schleiferi* isolates. In addition, when resistance to more than three classes of antibiotics was considered, 71/158 (44.9 %) isolates were MDR-SS with an overall resistance to 5.5 (median) classes of antibiotic (range: 3–8) (Table S1). Over the time period studied the percentage of resistance to other antibiotics ranged from 7±7 % for chloramphenicol to 61±18 % for fluoroquinolones (Table 2). Rifampin was the exception, with only one isolate determined to be resistant over the 10-year period.

**Table 2.** Percentages of resistance to the different classes of antibiotic analysed for the 159 canine isolates of *Staphylococcus schleiferi
*

	MR-SS (PBP2a)	MR-SS (OXA)	MDR-SS	Macrolides	Lincosamides	Phenicols	Tetracyclines	Aminoglycosides	Fluoroquinolones	TMS	Rifampin
2020 (*n*=12)	36	57	21	11	21	0	0	0	54	0	8
2019 (*n*=23)	45	65	30	11	22	17	0	4	54	17	0
2018 (*n*=24)	52	50	29	6	13	0	0	13	82	4	0
2017 (*n*=18)	35	39	28	8	17	15	0	17	42	17	0
2016 (*n*=22)	86	95	68	16	32	9	0	18	77	23	0
2015 (*n*=17)	69	71	41	3	12	6	0	29	44	6	0
2014 (*n*=24)	58	67	50	17	33	13	0	42	58	25	0
2013* (*n*=2)	50	50	0	25	0	0	0	25	50	0	0
2012* (*n*=1)	100	100	100	50	100	0	0	50	100	100	0
2011 (*n*=6)	50	50	67	33	67	17	0	33	50	17	0
2010 (*n*=10)	100	70	30	5	10	0	0	16	60	0	0

*Very few isolates with no statistical relevance.

MDR-SS, multidrug-resistant SS; MR-SS, methicillin-resistant SS; OXA, oxacillin; PBP2a, penicillin-binding protein 2a; SS, *Staphylococcus schleiferi *; TMS, trimethoprim/sulfamethoxazole.

Compared with 2010, a significant increase in methicillin resistance (PBP2a or OXA) or multidrug resistance status was not seen for *S. schleiferi *. When the relationship of resistance prevalence with time was assessed, there was not any significant correlation between time and any antimicrobial evaluated (Fig. S2). In the multivariable analysis, the odds of having MDR-SS were eight times higher in dogs with MR-SS (OXA) (OR: 8.8; 95 % CI: 3.6–21.1; *P<*0.0001) when compared with dogs with non-MR-SS (OXA).

#### S. aureus

A total of 14 canine medical records with at least 1 positive culture for *S. aureus* were retrieved. Of the dogs cultured, eight were male and six were female. The age at the time of culture had an overall median of 6 years (range: 1–12 years). Because of the small number of records retrieved, the isolates were analysed descriptively, altogether as a group. Data on the PBP2a were available for 13 isolates (92.8%), cefoxitin was tested in 8 isolates (57%), whereas OXA was tested for all the isolates (100%) (Table S1). A total of 11/14 (78.6 %) cultures had more than 1 organism isolated. A total of 14/14 (100 %) and 9/13 (69.2 %) records indicated exposure to a systemic antibiotic or an anti-inflammatory medication (i.e. steroids, oclacitinib, cyclosporine, lokivetmab), respectively, in the year prior to culture.

In contrast to *S. pseudintermedius* and *S. schleiferi *, more concordance between PBP2a agglutination test [4/13 (30.7 %)] and OXA resistance [4/14 (28.6 %)] was seen for *S. aureus* isolates. When resistance patterns for OXA and cefoxitin were compared to each other, for the eight isolates tested for cefoxitin, seven out of eight (87.5%) had concordant results. Only one isolate susceptible to OXA was resistant to cefoxitin. Thus, the percentage of methicillin resistance for *S. aureus* using cefoxitin (3/8 isolates; 37.5%) was comparable to the percentage using PBP2a or OXA as references (Kruskal–Wallis: *P*>0.99). Similarly to *S. pseudintermedius* and *S. schleiferi *, a very strong correlation between PBP2a and OXA (r: 1; *P*<0.0001), but not with cefoxitin (*P*=0.1), was found for canine *S. aureus* isolates. In addition, when resistance to more than 3 classes of antibiotics was considered, 5/14 (14.3 %) isolates were MDR-SA with an overall resistance to 4 (median) classes of antibiotics (range: 3–6) (Table S1). The degree of resistance to other antibiotics ranged from 9 % for chloramphenicol to 25 % for tetracyclines. Rifampin was the exception, with no resistance detected over the 10-year period.

### Cats

#### S. pseudintermedius

A total of nine feline medical records with at least one positive culture for *S. pseudintermedius* were retrieved. Of the cats cultured, seven were male and two were female. The age at the time of culture had an overall median of 7 years (range: 2–12 years). Because of the small number of records retrieved, the isolates were analysed descriptively, altogether as a group. Data on the PBP2a were available for 6/9 isolates (66.6%), whereas OXA was tested for all the isolates (100%) (Table S1). A total of 6/9 (66.6 %) cultures had more than one organism isolated. A total of 9/9 (100 %) and 3/9 (33.3 %) records indicated exposure to a systemic antibiotic or an anti-inflammatory medication (i.e. steroids and oclacitinib), respectively, in the year prior to culture.

The percentage of methicillin resistance varied slightly based on the detection of the presence of PBP2a or OXA resistance alone. In fact, 6/6 (100 %) isolates tested were positive for the PBP2a agglutination test and 6/9 (66.6 %) showed OXA resistance. In addition, when resistance to more than three classes of antibiotics was considered, 7/9 (77.7 %) isolates were MDR-SP, with an overall resistance to seven (median) classes of antibiotics (range: 5–7) (Table S1). The degree of resistance to other antibiotics ranged from a median of 12.5 % for chloramphenicol to 87.5 % for fluoroquinolones. Rifampin was the exception, with no resistance detected over the 10-year period.

#### S. schleiferi

Only one feline medical record with at least one positive culture for *S. schleiferi* was retrieved. The cat cultured was an 11-year-old male cat. The isolate was positive for PBP2a and resistant to OXA (Table S1). The isolate was also resistant to four classes of antibiotics (MDR-SS*)* (Table S1). No other co-organisms were isolated. The record also indicated exposure to a systemic antibiotic but not to an anti-inflammatory medication in the year prior to culture. The isolate was susceptible to aminoglycosides, chloramphenicol, rifampin and TMS.

#### *S *. *aureus*

A total of 12 feline medical records with at least 1 positive culture for *S. aureus* were retrieved. Of the cats cultured, seven were male and five were female. The age at the time of culture had an overall median of 5 years (range: 0.5–19 years). Because of the small number of records retrieved, isolates were analysed descriptively, altogether as a group. Data on the PBP2a were available for 10/12 isolates (83.3 %), cefoxitin was tested in 5 isolates (41.7 %), whereas OXA was tested for all the isolates (100 %) (Table S1). A total of 7/12 (58.3 %) cultures had more than 1 organism isolated. A total of 11/12 (91.7 %) and 9/12 (75 %) records indicated exposure to a systemic antibiotic or an anti-inflammatory medication (i.e. steroids and oclacitinib), respectively, in the year prior to culture.

The percentage of methicillin resistance slightly varied based on the detection of the presence of PBP2a or OXA resistance alone. In fact, 1/10 (10 %) isolates were positive for the PBP2a agglutination test and 3/12 (25 %) showed OXA resistance. In addition, none of the isolates were considered to be MDR (Table S1). The degree of resistance to other classes of antibiotic ranged from a median of 16.6 % for clindamycin to 41.7 % for macrolides. No resistance to chloramphenicol, aminoglycosides, rifampin and TMS was demonstrated over the 10-year period.

When resistance patterns between OXA and cefoxitin were compared, all five isolates tested resistant for cefoxitin (100 %) were also resistant for OXA. However, even if the percentage of methicillin resistance for *S. aureus* using the cefoxitin (0/5 isolates; 0 %) was much lower than using PBP2a or OXA as references, a significant difference among the three references was not seen (Kruskal–Wallis: *P*>0.4).

## Discussion

The results of this study confirmed that *S. pseudintermedius* is the most common (76.9 %) CPS retrieved from dogs with cutaneous infections in a tertiary referral clinic. This was followed by *S. schleiferi* (21.2 %) and only a few cases of *S. aureus* (1.9 %) were identified. *S. aureus* (54.5 %) followed by *S. pseudintermedius* (40.9 %) were the most represented CPS in feline dermatological patients in this study. Consequently, this study confirmed the higher incidence of *S. pseudintermedius* and *S. schleiferi* identified in dogs compared with cats. Out of 584 *S. pseudintermedius* and 160 *S. schleiferi* cultures, only 9 (1.5 %) and 1 (0.6 %), respectively, were isolated from cats. However, the isolation of *S. aureus* was similar between the two species, with 46 % of isolates harvested from cats and 54 % of isolates cultured from dogs.

The higher prevalence of *S. pseudintermedius* and *S. schleiferi* in canine patients was expected and it agrees with the recently highlighted clinical consensus guidelines [10, 24, 25]. Although a direct geographical influence has been suggested [10], the prevalence of canine MR staphylococci in this study is also in accordance with the previously reported epidemiological studies [10, 24, 25]. Although less is known about the true prevalence of staphylococcal skin infection in cats, the results of this study are in line with a previous report showing an incidence of 6 % *S. pseudintermedius* from feline bacterial cultures submitted to two microbiology laboratories [17]. Similar to what has previously been reported in Germany [17], this study suggests a higher prevalence of MR-SP and a much lower prevalence of MRSA in both species. Nevertheless, these data disagree with previous studies reporting MR-SP in up to 7.5 % of cats presenting to veterinary hospitals for inflammatory skin diseases or infections [10, 26, 27]. In this study the prevalence of MR-SP in cats ranged between 66.6 and 100 % based on the test used, PBP2a versus OXA.

The results of this study highlight how the antimicrobial patterns of cutaneous staphylococcal infections have changed in the past 10 years. In particular, in the canine population analysed here, an increase in antibiotic resistance has been seen for chloramphenicol. This reflects the increased use of an antibiotic that was once rarely used. These results are somewhat in agreement with a previous study published a few years ago in the UK [16]. In that study the authors reported an overall incidence of resistance below 10 %, in contrast to this study, in which the percentage of resistance ranged from 13 % for chloramphenicol to 52 % for lincosamides. Similar to the present study, an increasing trend of resistance for β-lactam antibiotics (ampicillin/amoxicillin, cefovecin, cephalexin) was also reported [16]. Although a significant increase in clindamycin, enrofloxacin and TMS has not been previously reported [16].

Most likely the change in antibiotic usage has been dictated by the increasing prevalence of MDR infection in clinical practice. This change in antibiotic pressure is evident in the results of this study, in which almost 50 % of the *S. pseudintermedius* isolates retrieved were resistant to β-lactams, macrolides, lincosamides, or fluoroquinolones. This resistance leaves clinicians without any reasonable antibiotic choices other than chloramphenicol, TMS or rifampin. The use of tetracyclines has also increased over the past 10 years, a phenomenon potentially caused by the decrease in the cost of doxycycline and minocycline combined with the lack of other antibiotic options. An increase in resistance to tetracyclines was not seen in this cohort, probably because of the changes in CLSI breakpoints for tetracycline antibiotics over time. In addition, the test for minocycline and tetracycline was not added to the sensitivity plates until 2017. For many isolates sensitivity results were NI for tetracyclines. This limits the detection of any resistance trend over time for this class of antibiotics. Notably, antibiotic resistance to rifampin was extremely rare (1/389 *S. pseudintermedius* and 1/128 *S. schleiferi* isolates) in this cohort of dogs, reflecting the continued rare use of such antibiotics in veterinary medicine.

An increase in canine MR-SP, but not in MR-SS, was also noted when referring to the methicillin resistance status in terms of OXA resistance. The percentage of MR-SP (OXA) increased from 28–80 % over the past 10 years. However, such changes were not observed when considering the detection of PBP2a – the preferred method for identification of MR-SP isolates [10]. It is also possible that the observed increased prevalence of OXA-resistant isolates could be due to the change in sensitivity breakpoints released by the CLSI. A decrease in the breakpoint would lead to the misidentification of susceptible isolates, whereas they would have been classified as resistant if the most current breakpoints were used. It was surprising that the trend analysis did not show an increase in multidrug resistance prevalence over the years based on the degree of resistance to antibiotics reported. It was also interesting to note that canine MDR-SP are generally resistant to more classes of antibiotics than MRSA (six versus four).

Of interest were the results of this study showing how the presence of methicillin resistance status, as well as the exposure to antibiotics within the year prior to culture, were both identified as major risk factors for the insurgence of MDR-SP (three times higher). By contrast, only the presence of methicillin resistance status was identified as a major risk factor for MDR-SS.

The ability of these bacteria to transfer genes between one another makes resistance in staphylococci a pressing public health concern [8, 9]. The ability of these pathogens to linger on healthy skin and in the environment increases the probability of reinfection and zoonotic transmission [6]. As MR-SP skin infections have increased in companion animals, MRSA skin infections have also increased in humans [4, 5]. Previous studies have identified identical strains of MR-SP and MRSA colonizing humans and their pets [4, 6]. Furthermore, human and veterinary patients afflicted with these infections sometimes share the same treatments. One study found that an increasing number of MRSA and MR-SP infections, in humans and companion animals respectively, are no longer susceptible to the widely used topical antibiotic mupirocin [28]. This has inspired debates concerning whether treatments such as these should be limited to human use. However, it is necessary to combine limitations on antibiotic use with additional strategies to control the proliferation of these resistant strains [28].

A regression analysis was applied to the canine data from this study to evaluate the risk factors associated with multidrug resistance status. It is interesting to note that both methicillin resistance (OXA) and methicillin resistance (PBP2a) status were highly associated with multidrug resistance for *S. pseudintermedius *, but only methicillin resistance (OXA) was associated with multidrug resistance for *S. schleiferi *. This is consistent with findings from previous studies showing the ability of MR strains to also obtain multidrug resistance genes through other genetic mechanisms [4, 5]. Similarly, exposure to systemic antibiotics in the year prior to culture was also considered a high risk for multidrug resistance in both *S. pseudintermedius* and *S. schleiferi *. These data are somewhat expected and in agreement with previous studies [29–31]. In particular, one epidemiological study isolated MR-SP from dogs following systemic antibiotic use when these dogs did not have MR-SP infection prior to medication administration [12].

A likely contributing factor to the switch in antibiotic resistance pattern could be the disruption of the normal cutaneous microbiota by the use of broad-spectrum antibiotics. This would increase the selection pressure favouring the insurgence of antibiotic-resistant isolates of staphylococci [4]. This phenomenon has encouraged practitioners to utilize topical antiseptic treatments whenever possible, especially for localized or mild infections [4]. Another important consideration is the role that underlying conditions play in the predisposition to develop these resistant infections. Certain conditions, such as atopic dermatitis, can also alter the normal microbiota of the skin [6]. Timely diagnosis and treatment for underlying conditions can limit the cycles of antibiotics utilized while attempting to gain control of secondary skin infections [6].

In this study, both sensitivity to OXA and positivity for PBP2a were used as parameters to determine the methicillin resistance status of the isolates. Cefoxitin was also considered for *S. aureus *. For *S. pseudintermedius *, there was a strong correlation between OXA and PBP2a status, with only 6 % of the isolates showing discordant results. Likewise, a strong correlation between OXA and PBP2a status was also seen for *S. schleiferi *, with only 0.6 % of the isolates having discordant results. This discordance can be due to many factors, including, but not limited to, the heterogenous expression of the mecA gene in bacterial cells leading to a negative PBP2a agglutination test [32], or the production of an inactive PBP2a translated from a truncated *mecA* gene [33], or even from a failure to produce any PBP2a protein because of a mutation in the *mecA* gene [33].

Regarding *S. aureus *, a perfect correlation between OXA and PBP2a status was seen, with no discordant results between the two parameters when both were present for the same isolate. However, a lack of significant correlation was seen between cefoxitin and the OXA and/or the PBP2a status. These results indicate how, although the identification of the *mecA* gene would be ideal for *S. pseudintermedius* and *S. schleiferi *, the use of OXA and PBP2a could potentially be interchangeable for the definition of methicillin resistance status as previously suggested [33]. Very few isolates showed a discordance between PBP2a and OXA susceptibility.

It is worth reminding that none of the tests for *mecA*, PBP2a, or OXA would be able to correctly identify CPS carrying the *mecC* gene [34]. In this study, a total of 337 *S. pseudintermedius* isolates were tested for cefoxitin, and of those only 5 (1.5 %) and 12 (3.6 %) isolates had a discordant result with OXA or PBP2a, respectively. No isolate showed a discordant result between cefoxitin and OXA and PBP2a together. Although, recent studies have shown a lack of detection of *mecC* in canine MR-SP isolates [35, 36], more studies are needed to better characterize the incidence of *mecC* in CPS in dogs and cats.

Unfortunately, few feline records were retrieved over the period analysed, making any inferences with such data very cautious. It was made clear that feline dermatological patients are much less commonly cultured compared with canine patients. When cultured, *S. aureus* and *S. pseudintermedius* are the most commonly isolated CPS in feline dermatological patients. It was also interesting to note that most of the feline *S. pseudintermedius* isolates were MR (up to 100 %) and MDR (77 %), a much higher prevalence than that seen with the canine counterparts. An overall prevalence of 77 and 87.5 % of antibiotic resistance was noted for lincosamides and fluoroquinolones, respectively. By contrast, only up to 25 % of *S. aureus* were MR and only 16.6 % were MDR. The reason for this discrepancy in resistance patterns between *S. pseudintermedius* and *S. aureus* is not clear.

A significant limitation of this study is the bias toward dermatological patients. Selection of cultures from this group of patients increased the likelihood of isolating the desired staphylococcal infections to be examined. Compared to healthy patients, patients referred to the dermatology service are more likely present with recurrent infections and a history of previous antibiotic use. Another significant limitation of this study is the change over time in the CLSI’s clinical breakpoints for staphylococci. Such changes were not considered in this study because of the lack of minimum inhibitory concentrations reported in the sensitivity panels over the years making the appropriate correction impossible. An additional limitation of the study is the fact that these data only reflect the epidemiological scenario in one location; a multicentric study would have been beneficial in capturing a more global scenario. Finally, in this study the urease and/or the coagulase status was not reported for *S. schleiferi* in the records. Therefore, the determination of *S. coagulans* versus *S. schleiferi* was not possible [37]. As a result, both species were grouped under the umbrella of *S. schleiferi *.

### Conclusions

The results of this study suggest an increase in antibiotic-specific resistance in dermatological patients seen in a tertiary unit. Such a trend is mainly evident for older antibiotics (i.e. chloramphenicol) with significant side effects that have regained popularity because of reduced sensitivity to more ‘benign’ antibiotics. In addition, the data from this study highlight how the methicillin resistance status and the use of antibiotic within the year prior culture are significant factors in the selection of MDR-SP. Only the methicillin resistance status was identified as a major risk factor for MDR-SS.

This is also the first cohort that identified the incidence of CPS in feline patients seen in a referral dermatological unit. The data from this study suggest that *S. aureus* is the most common CPS isolated from feline dermatological patients, followed by *S. pseudintermedius *, with the latter being most commonly MR.

As healthcare professions move forward, it is mandatory to use and implement as much as possible alternative therapies, hygiene guidelines, surveillance and education in the treatment of these infections. In areas with low levels of methicillin resistance, empirical treatments with first-tier systemic antibiotics should be preferred while culture and sensitivity results are pending. Monitoring the incidence of methicillin resistance and multidrug resistance across different locations is useful to compare trends, identify areas with prominent patterns of resistance and act accordingly.

## Supplementary Data

Supplementary material 1
