# Supplementary material for: Prevalence of multidrug-resistant coagulase-positive staphylococci in canine and feline dermatological patients over a 10-year period: a retrospective study
Source: Microbiology (Reading). 2023 Feb 14;169(2):001300. doi: 10.1099/mic.0.001300 (PMC10197874; doi:10.1099/mic.0.001300)
Supplement: Supplementary material 1 [file mic-169-1300-s001.pdf]

Table S1: Summary of the resistance status of the coagulase positive staphylococci identified in this study.

Animal species	Bacterial species	Number of isolates	Specimen	OX status	PBP2a status	MDR status
Dog	SP	575	Skin	56.2%	55.3%	52.8%
	SS	159	Skin	62.9%	56.8%	44.9%
	SA	14	Skin	100%	92.8%	14.3%
Cat	SP	9	Skin	100%	66.6%	77.7%
	SS	1	Skin	100%	100%	100%
	SA	12	Skin	100%	83.3%	0%

Table S2: Other Staphylococci identified in dogs and cats during 2010-2020

Dogs

Coagulase negative *Staphylococcus* group	2
Staphylococcus beta hemolytic	7
Staphylococcus epidermidis	5
Staphylococcus lugdunensis	1
Staphylococcus spp.	35
Staphylococcus xylosus	2
Staphylococcus warneri	1

Cats

Staphylococcus beta hemolytic	3
Staphylococcus hyicus	4
Staphylococcus spp.	5

Figure S1: Percentage of resistance over time to the different classes of antibiotic analysed for the 575 canine isolates of *Staphylococcus pseudintermedius*. SP: *Staphylococcus pseudintermedius*; MRSP: Methicillin-resistant SP; MDR-SP: Multidrug resistant SP; PBP2a: Penicillin binding protein 2a.

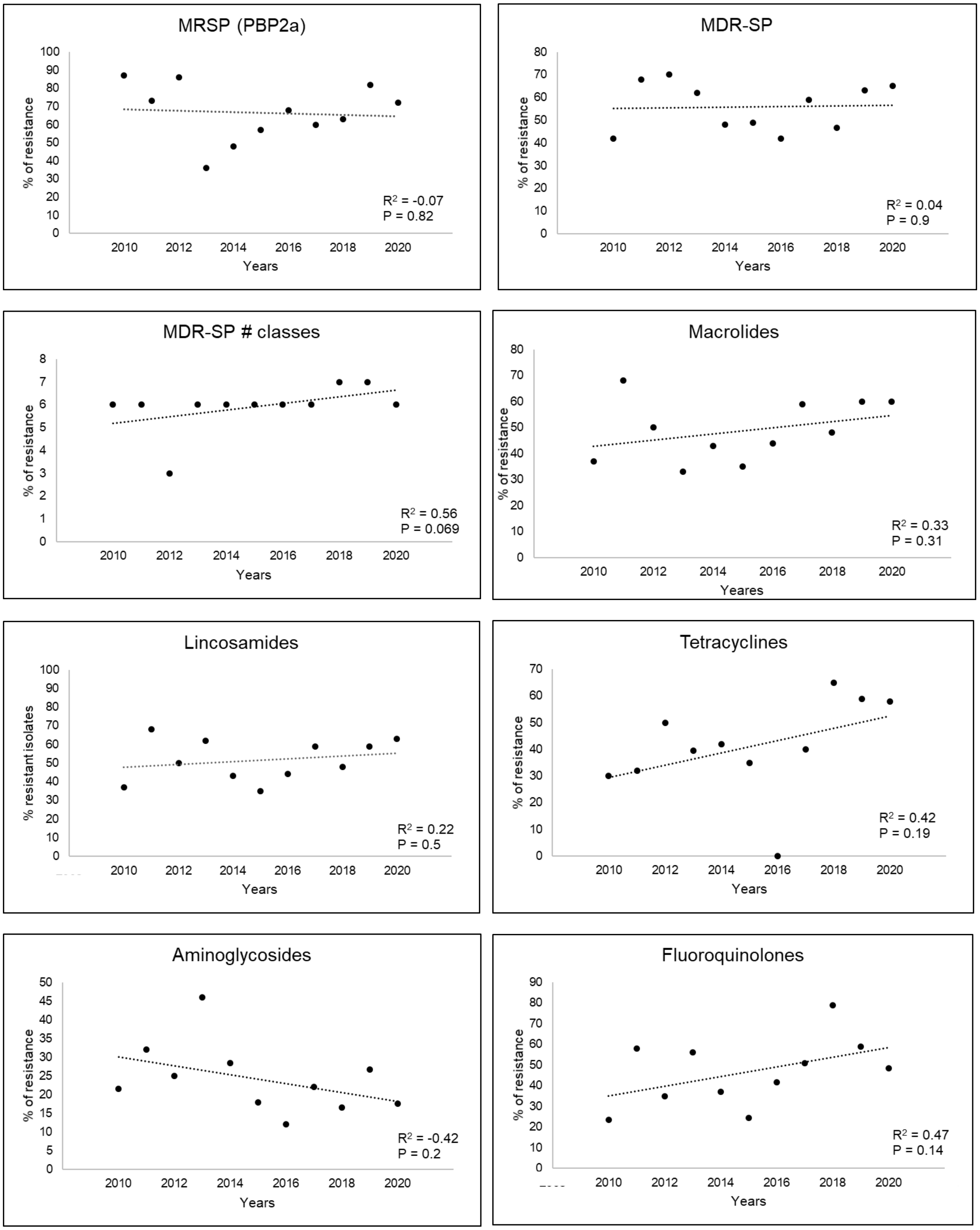

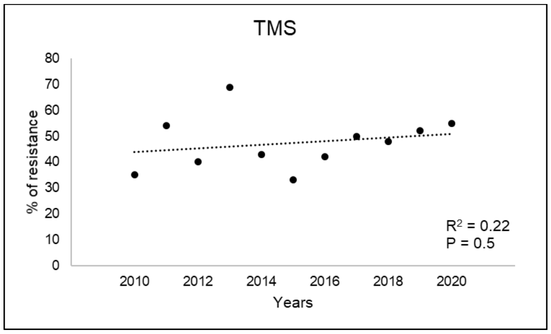
TMS
80
70
60
50
40
30
20
10
0
% of resistance
2010
2012
2014
2016
2018
2020
Years
R² = 0.22
P = 0.5

Figure S2: Percentage of resistance over time to the different classes of antibiotic analysed for the 159 canine isolates of *Staphylococcus schleiferi*. SS: *Staphylococcus schleiferi*; MR-SS: Methicillin-resistant SS; MDR-SS: Multidrug resistant SS; PBP2a: Penicillin binding protein 2a; OXA: oxacillin.

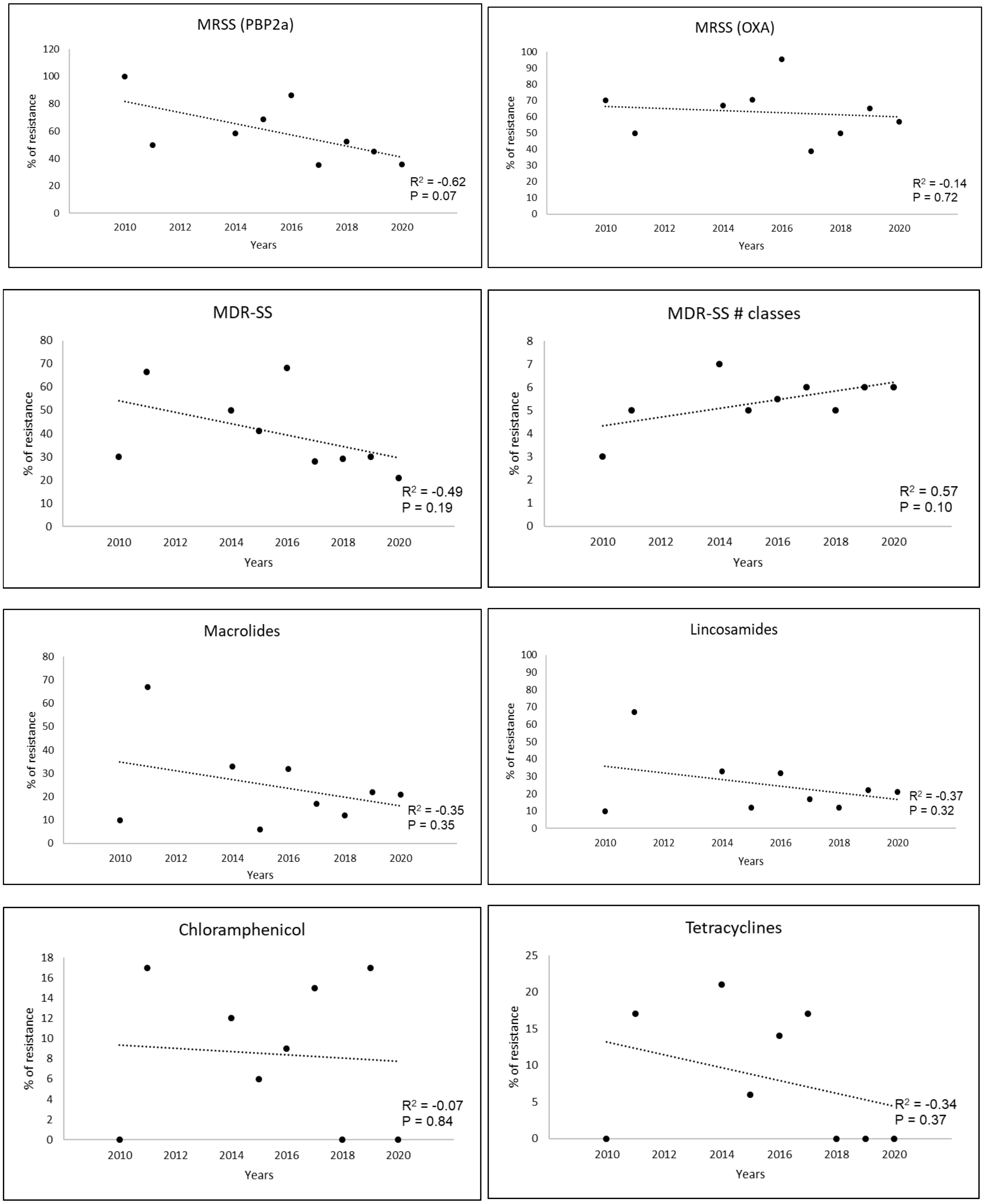

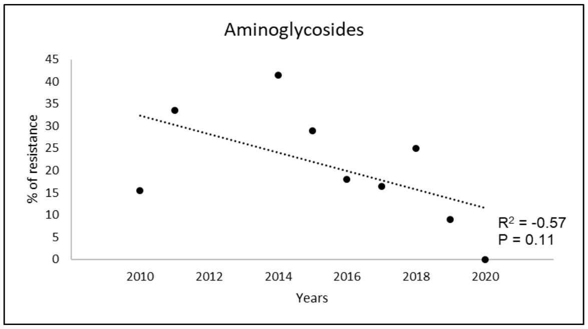
Aminoglycosides
% of resistance
Years
R2 = -0.57
P = 0.11

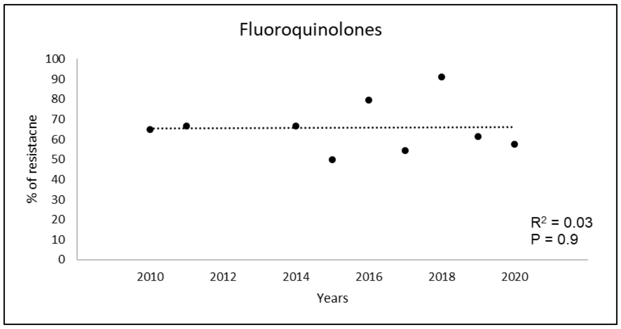
Fluoroquinolones
% of resistacne
Years
R2 = 0.03
P = 0.9

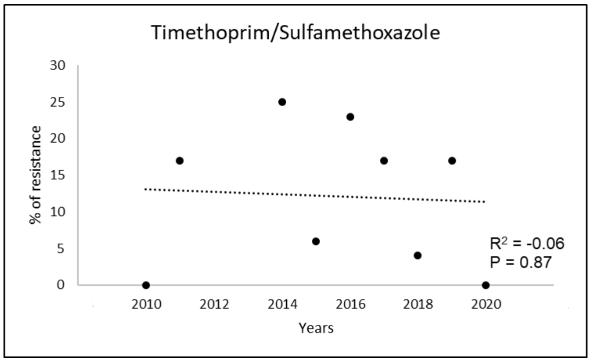
Timethoprim/Sulfamethoxazole
% of resistance
Years
R2 = -0.06
P = 0.87
